# Poetry from Pindar

**Published:** 1856-04

**Authors:** 


We know of no better illustration of the mode in which quacks and quack remedies sometimes gain reputation, than the case poetically reported by Peter Pinder, Esq., quoted below. True, this case has the advantage of ten thousand others, proclaimed by the proprietors of nostrums, as cured by their wonderful remedies, in that it has, at least, the credit of a probable coincidence, whilst those thousands are generally without even the color of truth, imparted by the evidence of coincidence, but are false from beginning to end.

Peter's Doctor has failed to report the instances of *failure*, where the pills were sent ass hunting, but in this his doctor is not unlike his posterity of humbuggers. They send their nostrums disease-hunting through the channels of their confiding victims, and commend them to their patients, upon the ground which caused our grand-mothers to administer calomel, "that it is a very "sarching" medicine." If nature be so fortunate as to resist the malady in spite of their remedies, the nostrum does the work; if they fail, it is for want of another box or bottle, which follows in quick succession, until in despair, the poor invalid turns for relief, when too late, to the regular profession, in whose hands he dies, and to whom he leaves the valuable legacy of the credit of having killed him.

A fellow, troubled with the itch
(Like Courtier-men) of getting rich;
And learning that a Doctor (*not a quack*,)
By means of a most potent pill,
Did verily and truly fill
Full many a time with gold his sack;
Resolved, by pill, to make a fortune too:
So set about it without more ado.

Hoist but the standard, folks will come,
With Heads as empty as a Drum,
The Quack puffs off his pill: none doubt him;
And numbers quickly flock'd about him.

A Bumkin came among the rest,
And thus the Man of Pill addrest:
"Zur, hearing what is come to pass;
That your fine pill hath cured the King,
And able to do every thing;
D'ye think, Zur, that 'twill make me *vind my ass?*
I've lost my Ass, Zur, zo should like to try it:
If this be your opinion, Zur, I'll buy it."—

"*Undoubtedly*," the Quack replied:
"Yes, Master Hob, it *should* be *tried*."
Then down Hob's gullet, cure or kill,
The grand imposter push'd the pill.

Hob paid his fee, and off he went;
And, traveling on about an hour,
His *bowels* sore with pains were rent:
Such was the pill's *surprising* pow'r.

No longer able to *contain,*
Hob in a hurry left the lane
(How *decent!* what can *decency* surpass?),
And sought the *grove;* where Hob's two eyes,
Wide staring, saw with huge *surprise*
His long-eared servant Jack, his *ass.*
Ye Gods, how *happy* was the meeting!
Hob kissing Jack; and Jack, Hob greeting.

"Adzooks, a *lucky* pill!" quoth Hob:
Yes, yes, the pill hath *done the job.*"—
*Pill* grew the subject of the Village tattle:
At last it gain'd a heap of fame;
Not only good for *blind* and *lame,*
But good too for recovering all *stray'd cattle.*

